# Different Transcriptomic Response to *T. cruzi* Infection in hiPSC-Derived Cardiomyocytes From Chagas Disease Patients With and Without Chronic Cardiomyopathy

**DOI:** 10.3389/fcimb.2022.904747

**Published:** 2022-07-07

**Authors:** Theo G. M. Oliveira, Gabriela Venturini, Juliana M. Alvim, Larissa L. Feijó, Carla L. Dinardo, Ester C. Sabino, Jonathan G. Seidman, Christine E. Seidman, Jose E. Krieger, Alexandre C. Pereira

**Affiliations:** ^1^ Laboratório de Genética e Cardiologia Molecular, Instituto do Coração (InCor), Hospital das Clínicas, Faculdade de Medicina, Universidade de São Paulo (HC/FMUSP), São Paulo, Brazil; ^2^ Instituto do Coração (InCor), Hospital das Clínicas, Faculdade de Medicina, Universidade de São Paulo (HC/FMUSP), São Paulo, Brazil; ^3^ Fundação Pró-Sangue Hemocentro de São Paulo, Divisão de Pesquisa – São Paulo, SP, Brazil; ^4^ Genetics Department, Harvard Medical School, MA, United States; ^5^ Instituto do Medicina Tropical (IMT), Universidade de São Paulo – São Paulo, SP, Brazil; ^6^ Cardiovascular Division, Brigham and Women’s Hospital, & Harvard Medical School, Boston, MA, United States; ^7^ Howard Hughes Medical Institute, Chevy Chase, MD, United States

**Keywords:** chagas disease, chagas cardiomyopathy, iPSC (induced pluripotent stem cell), *Trypanosoma cruzi (T. cruzi*), RNA-Seq, cardiomyocytes (CMs)

## Abstract

Chagas disease is a tropical zoonosis caused by *Trypanosoma cruzi*. After infection, the host present an acute phase, usually asymptomatic, in which an extensive parasite proliferation and intense innate immune activity occurs, followed by a chronic phase, characterized by low parasitemia and development of specific immunity. Most individuals in the chronic phase remain without symptoms or organ damage, a state called indeterminate IND form. However, 20 to 40% of individuals develop cardiac or gastrointestinal complications at any time in life. Cardiomyocytes have an important role in the development of Chronic Chagas Cardiomyopathy (CCC) due to transcriptional and metabolic alterations that are crucial for the parasite survival and replication. However, it still not clear why some infected individuals progress to a cardiomyopathy phase, while others remain asymptomatic. In this work, we used hiPSCs-derived cardiomyocytes (hiPSC-CM) to investigate patterns of infection, proliferation and transcriptional response in IND and CCC patients. Our data show that *T. cruzi* infection and proliferation efficiency do not differ significantly in PBMCs and hiPSC-CM from both groups. However, RNA-seq analysis in hiPSC-CM infected for 24 hours showed a significantly different transcriptional response to the parasite in cells from IND or CCC patients. Cardiomyocytes from IND showed significant differences in the expression of genes related to antigen processing and presentation, as well as, immune co-stimulatory molecules. Furthermore, the downregulation of collagen production genes and extracellular matrix components was significantly different in these cells. Cardiomyocytes from CCC, in turn, showed increased expression of mTORC1 pathway and unfolded protein response genes, both associated to increased intracellular ROS production. These data point to a differential pattern of response, determined by baseline genetic differences between groups, which may have an impact on the development of a chronic outcome with or without the presentation of cardiac symptoms.

## Introduction

Chagas disease (ChD), also referred as American trypanosomiasis, is a tropical anthropozoonosis traditionally endemic in rural areas of South and Central America where the transmission of *Trypanosoma cruzi* (*T. cruzi*) occurs through the contact with contaminated feces of Triatomine insects (also known as “kissing bugs”). Although the number of new cases and deaths has been decreasing since 1980’s, in 2010 ChD was still responsible for approximately 12.000 deaths per year in endemic countries (Lidani et al., 2019), where 6 million people live with the disease according to the Pan American Health Organization (PAHO – paho.org). In the United States, it is estimated that at least 300.000 people have ChD, most of them immigrants from endemic countries (Pérez-Molina and Molina, 2017).

Initially, ChD presents an acute phase in which *T. cruzi* proliferates in high amounts and its presence is easily detected in the blood stream. This phase is rarely symptomatic and can be taken as a common seasonal disease where fever and malaise are two common symptoms (Pérez-Molina and Molina, 2017). The acute phase is followed by the chronic phase in which the parasite vanishes from the circulation and IgG antibodies can be detected by serological tests. The most intriguing aspect of the chronic phase is that while 70% of individuals will remain in an asymptomatic indeterminate (IND) state for life, 30% will develop chronic Chagas cardiomyopathy (CCC) which is the most severe consequence of *T. cruzi* infection and affects individuals after a latency period of 10 to 30 years leading to late-stage heart failure, arrhythmias and thromboembolic events (Ribeiro et al., 2012). Curiously, it is still unclear how low intensity immune response, parasite persistence and cardiac tissue injury and remodeling interact to modulate the disease progression (Rassi et al., 2017).

The investigation of the transcriptional response to *T. cruzi* infection in non-phagocytic cells has been successful in describing the main pathways immediately disturbed by the parasite (ranging from 3 to 72 hours post infection or hpi) and the common responses between different cell types (Li et al., 2016). Within the first 24 hours of infection a broad-spectrum host transcriptional remodeling is important for parasite survival and replication. Cellular alterations such as increased mitochondrial oxidative metabolism, increased protein synthesis and cell cycle arrest were detected in human fibroblasts (Houston-Ludlam et al., 2016; Oliveira et al., 2020). Defense responses through type I interferon and chemokines production were repeatedly observed in fibroblasts, endothelial and smooth muscle cells (Costales et al., 2009) as well as in murine and human primary cardiomyocytes (Libisch et al., 2018).

Although transcriptomic studies using cardiac biopsies from chronic patients were important in defining the inflammatory landscape of CCC, these findings depict a long-term scenario in which many of the initial aspects of the response to the parasite infection cannot be determined. Furthermore, the limitation in obtaining cardiac biopsies, and the intrinsic distinction of heart function, from IND patients precludes a true comparative set-up which could reveal group-specific aspects of chronic ChD susceptibility. Similarly, murine or human cardiac cell lineages used in cellular models do not reveal patient-specific features, leaving important questions about the difference in cardiomyocyte transcriptional response between CCC and IND patients still unanswered.

In this study, we aimed to capture the early transcriptional responses to *T. cruzi* infection by generating human induced pluripotent stem-cell (hiPSC)-derived cardiomyocytes (hiPSC-CM) from both groups of ChD patients and re-infect these cells with the highly virulent *T. cruzi* Y strain for 24 hours, 30 days after differentiation. Our results show that in terms of infection capacity, *T. cruzi* can infect and replicate with the same efficiency inside PBMCs and hiPSC-CM of IND and CCC patients. Transcriptional responses in the first 24 hours of infection, however, revealed a different response against the parasite, with IND cardiomyocytes showing a more orchestrated pattern of innate immune activation and antigen presentation. These results bring new information regarding the capacity of patient-specific cardiomyocytes in dealing with the parasite invasion as well as setting a proper response to infection that is crucial to establish an early regulatory scenario which may have important implications in the chronic phase of ChD.

## Materials and Methods

### Ethics Statement and Patient Selection

This work was approved by the local ethics committee (Comissão de Ética para Análise de Projetos de Pesquisa – CAPPesq – CAAE: 89242218.0.0000.0068). Patients were selected from a cohort of the Instituto de Medicina Tropical (IMT – Universidade de São Paulo). We included patients with at least two confirmed serological tests for ChD. Indeterminate (IND) patients should present normal ECG and chest X-ray in at least two visits and chronic cardiac (CCC) patients should present cardiomegaly with ECG abnormalities in the absence or presence of left ventricle dysfunction (VEF<30%). Patients with previous history of myocardial infarction or any inherited cardiomyopathy were not included. All patients signed an informed consent. **Supplemental Table S1** shows age and sex information of included individuals as well as the main clinical features of CCC patients.

### PBMCs Extraction

Peripheral blood mononuclear cells (PBMCs) were extracted using Vacutainer^®^ CPT™ Tubes (BD) from five IND (IND1 to IND5) and six CCC (CCC1 to CCC6) patients. Briefly, 16 ml of blood were collected in two CPT tubes and centrifuged at 1800 x g for 40 minutes. After separation, the buffy coat was washed two times with PBS 1x and incubated for 15 minutes with erythrocyte lysing buffer. Cells were counted in a hemocytometer and 2.5 to 3x10^6^ PBMCs were frozen per vial.

### *Trypanosoma cruzi* Culture

The Y strain of *T.cruzi* modified to express GFP in the amastigote state (Ramirez et al., 2000) was gently provided by Prof. Sergio Schenkman (Universidade Federal de São Paulo – UNIFESP). *T. cruzi* trypomastigotes were co-cultured with LLC-MK2 cells (*Macaca mulatta*) in RPMI 10% FBS 1% PS in T175 flasks. The culture was daily monitored for the proliferation of intracellular parasites and media was changed every 72hs. New LLC-MK2 cells were added to the culture whenever needed. When high amounts of trypomastigotes were present, the supernatant was collected, centrifuged at 500 x g for 15 minutes and filtered in a 40µm cell strainer for *debris* removal. Trypomastigotes were harvested on the same day of infection assays.

### PBMCs Infection and Flow Cytometry Analysis

PBMCs were thawed and plated at 2 x10^4^ cells/well in 24-well plates with RPMI 10% FBS 1% PS and maintained at 37°C 5% CO_2_. A time course of infection was conducted with 5 time points (0, 3, 6, 24 and 48 hpi) in duplicate with a trypomastigote/cell ratio of 10:1 in a final volume of 1 mL. Time points 3, 6 and 24 hpi were defined as the infection period while the 48 hpi time point was defined as the proliferation period. Thus, after 24 hours of infection, the cells from the 48hpi time point were harvested and centrifuged for media change and removal of non-infecting parasites. Experiments were always conducted with one cardiac patient, one indeterminate patient and one non-chagasic control (CTRL) in parallel. A total of five experimental rounds were performed. After infection, cells were centrifuged in PBS 1x and labeled with anti-CD3 PE (T lymphocytes), anti-CD19 PECy5 (B lymphocytes) and anti-CD14 APC (monocytes) for 30 minutes at 4°C. Samples were analyzed in a BD Accuri™ C6 with a minimal threshold of 20.000 events inside the “classical” lymphocyte gate. The infection rate was calculated using the amount of GFP+ events and the replication rate was calculated with the mean intensity of GFP+ across time points. The replication rate between 24 and 48hpi was calculated as the 48/24hpi GFP intensity ratio. Results were generated in GraphPad Prism 8. ANOVA was used for three-group comparisons while T-test was used for pairwise comparisons. A p-value < 0.05 was considered as the statistical cutoff for significance.

### hiPSC Production

Human induced-pluripotent stem cells (hiPSC) from three CCC (CCC4, CCC5 and CCC6) and two IND (IND4 and IND5) patients were obtained through erythroblast reprogramming as previously reported (Chou et al., 2011; Dowey et al., 2012). The adjusted protocol was gently provided by Profª. Lygia da Veiga Pereira (Instituto de Biociências, IB/USP). Briefly, PBMCs were thawed and maintained in an erythroid enrichment media for 10 to 12 days. Media was composed of StemSpan (STEM CELL Technologies) supplemented with IGF-1 at 40ng/mL; SCF at 100ng/mL; IL-3 at 10ng/mL and EPO at 2U/mL (R&D systems). Dexamethasone was added at 1µM/mL for lymphocyte depletion. From D-12 to D-3 cells were cultivated at a density of 2x10^6^ cells/well in a 24-well plate. Usually on D-3, the culture was highly enriched for erythroblasts, so the density was changed to 1x10^6^ cells/well and cells were kept in a 6-well plate. When the density reached 2x10^6^ cells/well again, erythroblasts were directed to reprogramming (D0). A total of 1x10^6^ erythroblasts were nucleoporated with 1.25µg of EPI5™ Episomal iPSC Reprogramming Kit (Thermo Fisher) in a Nucleofector 2b (T-016 program) using the Human CD34+ Cell Nucleofector™ Kit (Lonza). At D2, media was changed to DMEM-High 10% FBS with GlutaMax^®^ (Thermo Fisher) and cells were transferred to one well of a 12-well plate containing GelTrex™ (Thermo Fisher) and were adhered to the matrix by centrifugation at 200 x g for 30 minutes. On D3, media was changed for E8 media (Thermo Fisher) supplemented with bFGF (20ng/mL) and Sodium Butirate (NaB, 0,25mM). From this point, E8 media was changed every other day. First colonies usually appeared between D8 and D12 after reprogramming. First colonies were picked around D15. At least three different clones with typical iPSC morphology were characterized for pluripotency markers with immunofluorescence and flow cytometry.

### Cardiomyocyte Differentiation

hiPSC-CM were obtained using the WNT-C59 protocol as previously reported (Sharma et al., 2018) with minor adjustments. Before starting the differentiation protocol, we prepared hiPSC by doing at least three long passages (1:15-20 split) followed by three short passages (1:2-3 split) with StemFlex media (Thermo Fisher) supplemented with 10 μM of rho kinase inhibitor (ROCKi). Cells were maintained in 6-well plates through the entire process. When confluence was around 80%, media was changed for RPMI B27- (RB-) supplemented with 9µM of CHIR99021 for 24 hours. The starting day was defined as Day 0 (D0). At D1 cells were washed with PBS 1x and fresh RB- was added to the culture (no CHIR99021). Cells were then kept untouched until D3 when RB- supplemented with 2µM of WNT-C59 (1:5000 dilution) was added to the wells. At D5 WNT-C59 was removed, cells were washed with PBS 1x and fresh RB- was added. At D7, RB- was changed for RPMI B27+ (RB+) with 1% PS. Beating hiPSC-CM were usually observed between D8 and D10. Once beating cells were observed in several spots of the well, RB+ with no glucose was added to the culture for non-cardiomyocyte removal. Glucose starving was maintained for 2 to 6 days (1 to 3 media changes), depending on the rate of cell death and visual inspection of non-beating or fibroblast-like cells. After the starving period, hiPSC-CM were maintained with RB+ 1% PS until D20, when cells were passaged with Trypsin (Thermo Fisher), filtered on a 100µm cell strainer, counted and evaluated for purity by the quantification of cardiac troponin through flow cytometry (**Supplemental Figure S4**). Cells were plated on 96-well plates at a density of 5x10^4^ cells/well and on 24-well plates at a density of 1x10^6^ cells/well for immunofluorescence and RNA-seq experiments, respectively.

### Immunofluorescence Analysis

An immunofluorescence assay was conducted in 5 time points (0, 3, 6, 24 and 48 hpi). hiPSC-CM were incubated with parasites in RB+ 1%PS in a parasite/cell ratio of 10:1. After the period of incubation, cells were washed with PBS 1x and fixed with PFA 4% for 20 minutes at room temperature. Nuclei were stained with DAPI and cells were analyzed in an EVOS™ M7000 Imaging System (Thermo Fisher) at the same day of fixation. Eight random fields of each well were captured and GFP+ amastigotes and stained nuclei were counted in a custom MATLAB (Mathworks Inc.) script developed by our group (data not published). The parasite/nuclei ratio was used as input variable for ANOVA in GraphPad Prism 8.

### RNA-Seq Experiment

Day 30 hiPSC-CM were infected with *T. cruzi* in a parasite/cell ratio of 10:1. Two infected and two non-infected replicates were generated per differentiated clone. RNA-seq replicates were maintained in contact with trypomastigotes in RB+ 1% PS for 24 hours at 37°C 5% CO_2_. Previous studies have shown that 24 hpi is enough for the Y strain to cause significant transcriptional modifications in human cardiomyocytes and other cell types (Houston-Ludlam et al., 2016; Bozzi et al., 2019). In addition, at 24 hpi no significant intracellular replication has taken place. At 24 hpi cells were washed twice with PBS 1x for extracellular parasites removal and lysed with TRIzol™ reagent (Thermo Fisher) for RNA extraction. Non-infected replicates were maintained in the same condition as infected ones. Library preparation was conducted using the Nextera library preparation method. RNA-Seq libraries were pooled and run on four lanes (one flow cell) using the Illumina NextSeq500 platform. Coverage calculation was adjusted to generate 30–50M reads per replicate. The raw reads were aligned by STAR to human genome (hg38).

### Differential Gene Expression and Pathway Enrichment Analysis

Differential gene expression (DE) analysis was conducted with DESeq2 (Love et al., 2014) package in RStudio. Genes with a sum of counts lower than 10 reads were considered as low count genes and were removed before analysis. DESeq2 object was designed considering differentiation batch, group (CCC or IND), time (0 or 24 hpi) and an interaction term for time and group. The IND group and 0 hpi were defined as reference levels. In the present analysis we were particularly interested in genes with a differential response between groups upon *T. cruzi* infection. Thus, we have selected genes with a significant interaction term p adjusted value (padj < 0.05).

Gene Set Enrichment Analysis (GSEA) was carried out with a gene list ranked by the formula |signal(log2FoldChange) x -log10(padj)| using the gene set enrichment (gse) function of ClusterProfiler (Wu et al., 2021) package. Heat maps were created with Morpheus web-tool (https://software.broadinstitute.org/morpheus) and KEGG pathways were plotted with the Pathview (Luo and Brouwer, 2013) package in Rstudio.

## Results

### *T. cruzi* Infection and Replication Efficiency in PBMCs and hiPSC-CM

**Figure 1:**
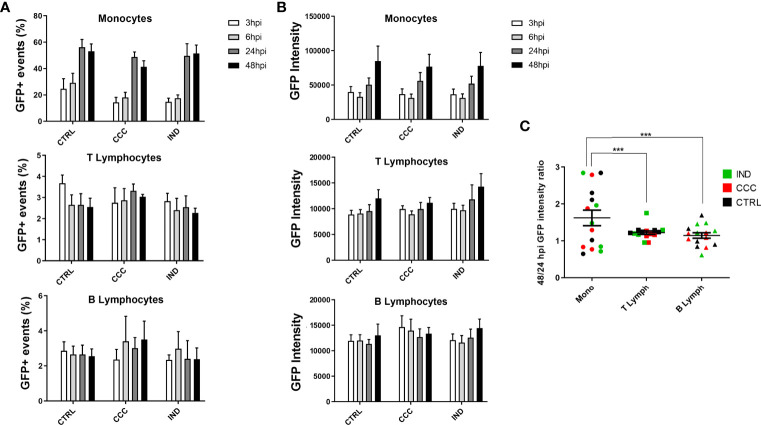
Results from infection assay of PBMCs by flow cytometry with GFP-expressing Y strain. **(A)** The infection rate over time points was analyzed through the percentage of GFP+ events in Monocytes, T and B Lymphocytes. **(B)** The proliferation rate over time points was analyzed through the GFP intensity across times points in Monocytes, T and B Lymphocytes. **(C)** The 48/24 hpi GFP intensity ratio was used to calculate the proliferation between 24 and 48 hpi in Monocytes, T and B Lymphocytes. (n = 5 CCC; 5 IND and 5 Non-infected controls). The symbol *** means P ≤ 0.001.

To establish whether *T. cruzi* infection efficiency was different between IND and CCC patients, we conducted time course infection experiments both in PBMCs and hiPSC-CM. The PBMC infection experiments were analyzed based on the following variables: percentage of GFP+ events over time (parasite infection), mean GFP intensity over time (parasite replication) and GFP 48/24 hpi intensity ratio (to measure the increase in GFP intensity between 24 and 48 hpi). To assure that the number of cells did not differ over time or by cell type, we compared the number of cells analyzed between groups and did not observe any statistical difference. It was possible to observe, however, that monocytes and B lymphocytes tend to diminish in number over time, while T lymphocytes remain practically constant (**Supplemental Figure S1**). Monocytes were the cell type with the highest percentage of GFP+ events over time (**Figure 1**, Panel A) reaching at certain points an infection rate above 50%, while T and B lymphocytes maintain a lower and constant rate of infection. When comparing the number of GFP+ events between the groups for each analyzed cell type it was not possible to detect a significant difference in any of the four time-points of infection.

When comparing the mean GFP intensity between groups for each cell type, it was again possible to observe that monocytes are the cell type with the greatest increase over time (**Figure 1B**), with a significant increase in the mean GFP intensity already at 24 hpi. Interestingly, T lymphocytes show an increasing tendency from 48 hpi onwards, which indicates a later increase in parasite replication compared to monocytes. Again, when comparing the GFP intensity between the groups for each analyzed cell type it was not possible to identify a significant difference at any time of infection. As for the 48/24 hpi GFP intensity ratio we did not observe a significant difference between groups, for any of the analyzed cell types (**Supplemental Figure S2**). However, when comparing the difference of this same variable considering only the cell type, we observed that monocytes have a significantly higher GFP intensity ratio of 48/24 hpi compared to T and B lymphocytes, suggesting different intracellular replication dynamics among these cell types (**Figure 1C**).

**Figure 2:**
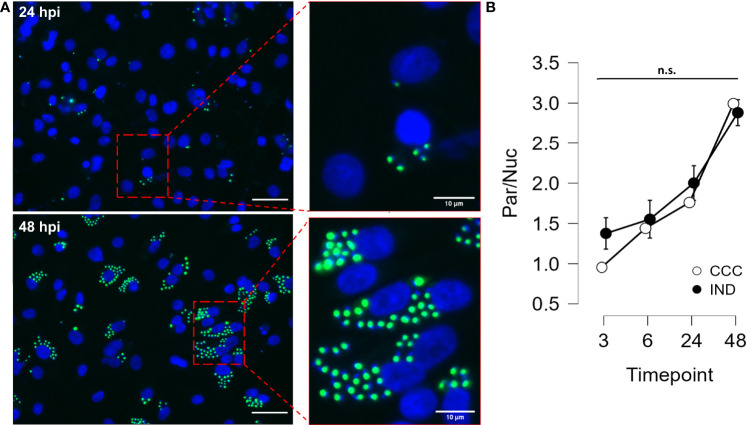
Results from hiPSC-CM infection assay with GFP-expressing Y strain. **(A)** hiPSC-CM were infected with *T. cruzi* Y strain modified to express GFP in the amastigote form. Cell nuclei were stained with DAPI (blue) (Left panel bars = 40µm). **(B)** Time course (3, 6, 24 and 48 hpi) analysis of infection in hiPSC-CM from IND and CCC patients. Nuclei and amastigotes were counted in a custom script and the Parasite/Nucleus ratio was compared along time points. (n = 3 CCC and 2 IND). n.s., not significant.

The same time-course experiment was performed in hiPSC-CM for both groups, with the readout defined as the number of intracellular amastigotes per cell nuclei. **Figure 2** shows how intracellular amastigotes were detected overtime in immunofluorescence images. Similar to what was used for PBMCs, the interval between 3 to 24 hpi was defined as the *T.cruzi* infection period and the interval between 24 to 48 hpi as the replication period. It was possible to detect GFP+ amastigotes already at 3 hpi and at 48 hpi most of nuclei were surrounded by highly GFP-expressing amastigotes, confirming the increment of GFP intensity overtime. After performing an ANOVA for detecting differences in the increasing parasite/cell ratio, no significant difference was observed between groups at any time point.

### RNA-Seq Results

**Figure 3:**
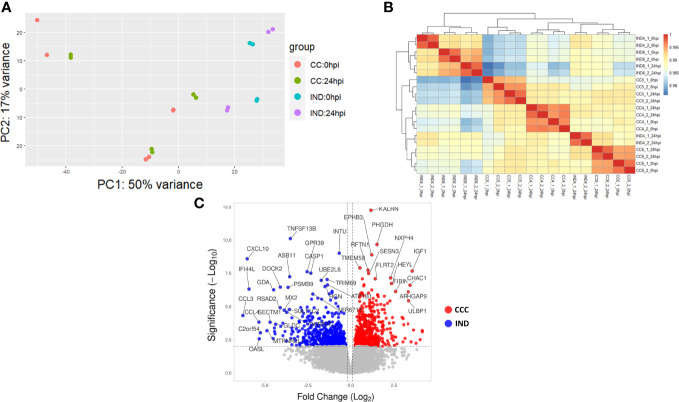
RNA-seq results. **(A)** PCA plot showing the variability among CCC and IND replicates at 0 and 24 hpi (CCC replicates = 6 CCC_0hpi, 6 CCC_24hpi. IND replicates = 4 IND_0hpi and 4 IND_24hpi). **(B)** Hierarchical clustering of replicates. **(C)** Volcano plot showing the L2FC distribution of DEGs in the interaction term output.

A total of 20 replicates (6 CCC_0hpi, 6 CCC_24hpi, 4 IND_0hpi and 4 IND_24hpi) were sequenced in a single run. In total, 577 million reads were generated with an average of 28.8 million reads per sample. Count distributions per sample are shown in **Supplemental Figure S3**. Reads were checked for quality and adaptor trimming with FASTQC before alignment and count generation. All samples passed QC steps and were used for further analysis. Principal component analysis (PCA) revealed a clone-related pattern of clustering. Interestingly, no differentiation batch effects were observed (**Figure 3A**). PC1 accounted for 50% of the sample’s variance, indicating that most of the variability among samples can be explained by phenotype group. Hierarchical clustering show that most clones tend to cluster together paired by time (**Figure 3B**).

### GSEA Reveal Group Specific Responses to Infection

In total, 463 DEGs with an adjusted p-value (padj) < 0.05 were selected from the interaction term (time:group) output after DE analysis (from now on we will refer to DEG as those with a significant interaction term). Of these, 395 (215 positive and 180 negative L2FC – **Figure 3C**) had a valid entrez ID and were used for further analysis.

**Figure 4:**
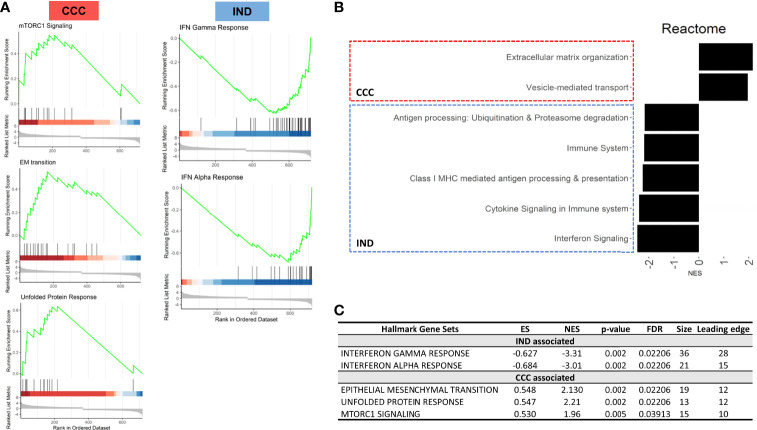
Results from GSEA with Hallmark terms. **(A)** GSEA plots showing the rank distribution of DEGs in Hallmark gene sets and the association with each phenotype (red bars – CCC; blue bars – IND). **(B)** Reactome plot with the NES for the pathways associated to each group. **(C)** Hallmark gene sets associated with each phenotype. ES, Enrichment Score; NES, Normalized enrichment score; FDR, False-discovery rate.

Gene set enrichment analysis (GSEA) was performed to check for group-specific enrichments. At first, we applied the padj <.05 DEGs list to GSEA with Hallmark [MSigDB collections (Liberzon et al., 2015)] gene sets, however no significant associations were found for any condition. Using a cutoff with padj < 0.1 we incremented our DEGs list to 719 genes with a valid entrez ID and ranked the list as mentioned in the methods section. We limited the analysis to a minimum and maximum set size of 5 and 500, respectively, and a maximum of 1000 permutations. Hallmark GSEA revealed three gene sets associated with the CCC group: EM transition (EMT), mTORC1 signaling and unfolded protein response (UPR). Two gene sets were associated with IND: Interferon (IFN) alpha response and gamma response. **Figure 4** shows GSEA plots for significant terms with its FDR values (panels A and C). In total, 28 genes with negative L2FC (IND) and 30 genes with positive L2FC (CCC) were associated to a gene set. **Supplemental Table S2** includes non-significant terms associated with each phenotype.

**Figure 5:**
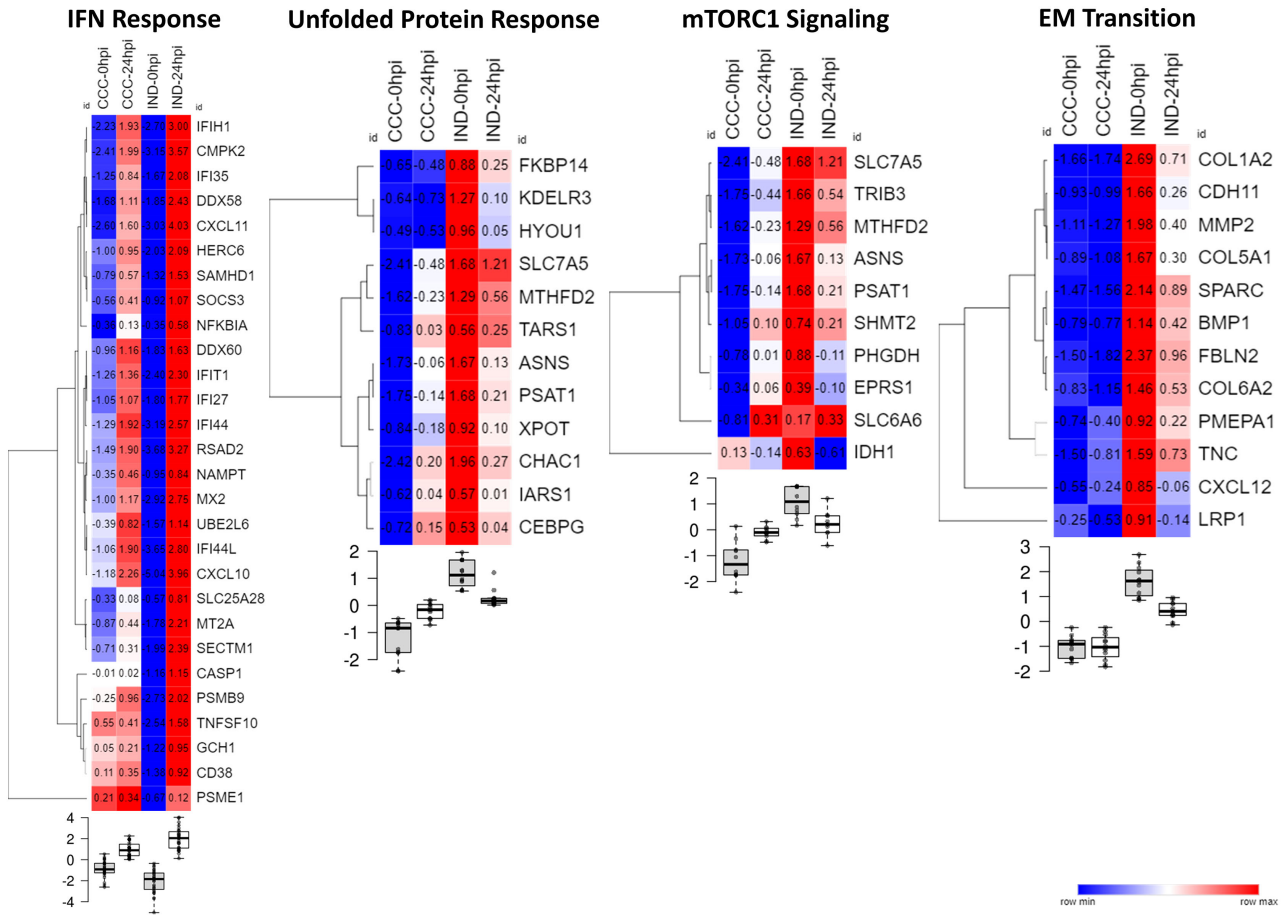
Heat maps showing the normalize expression of significant DEGs across conditions in their respective Hallmark gene set. Samples were normalized by the mean counts. Below each heat map the boxplots show the level of gene expression at every time point (0 or 24 hpi) for each group (CCC or IND).

**Figure 5** shows the expression of genes inside the enriched gene sets along the time course of infection. IFN response is a well-known early response to *T. cruzi* infection and, although both groups significantly upregulated IFN response genes the effect of infection resulted in a more acute shifting in the expression of these genes in the IND group. Conversely, all gene sets enriched in CCC suffered a significant downregulation in IND cardiomyocytes after infection. UPR and mTORC1 signaling were upregulated in CCC group as a response to infection while EMT remained stable from 0 to 24 hpi.

The gene list with significant DEGs (padj < 0.05) from the interaction term was submitted to GSEA with Reactome so that we could unravel more specific pathways by which enriched genes were acting. We applied the same parameters for set size and permutation used in the previous analysis. The pattern of association reproduced the Hallmark GSEA results, with immune-related pathways associated with IND and ECM and collagen production associated with CCC. There was an increment of 12 and 9 genes assigned to a pathway for IND and CCC, respectively (**Supplemental Figure S5**). Reactome specified not only the association of IFN related pathways with the IND phenotype, but also returned other important processes of immune-cells recruitment and establishment of adaptive immune response such as antigen processing (ubiquitination and proteasome degradation) and class MHC-I presentation. **Figure 4** shows the associated Reactome pathways (Panel B) with normalized enrichment score (NES). The connection plot of leading-edge genes can be found in **Supplemental Figure S6B**.

### CCC-Associated DEGs Include Key ER Stress Response and ECM Remodeling Genes

The unfolded protein response is mainly triggered by endoplasmic reticulum (ER) stress. Inside this gene set there were genes related to increase in protein translation (*TARS1*, *IARS1* and *ASNS*) as well as ER stress-mediated apoptosis (*CHAC1* and *CEBPG*). Collagen-production genes (*COL1A2*, *COL6A2, COL5A1* and *COLGALT2)* and other genes related to structural composition of ECM (*TNC*, *FBLN2*, *BMP1* and *MMP2*) are related both to “EM transition” (Hallmark) and “Extracellular-matrix organization” (Reactome). The gene sets “Vesicle-mediated transport” and “extracellular-matrix organization” were connected by *SPARC* and *COL1A2* genes (**Supplemental Figure S6B**), probably indicating vesicle mobilization either in the exporting of ECM components and in the vesicle-mediated entrance of *T. cruzi*. Concordantly, mTORC1 signaling genes up-regulation may contribute to these phenomena once these genes are involved in the formation of the parasitophorous vacuole, a crucial structure in *T.cruzi* internalization and proliferation.

### IND-Associated DEGs Include Chemokines, HLA-Coding Genes and Co-Stimulatory Molecules

**Figure 6:**
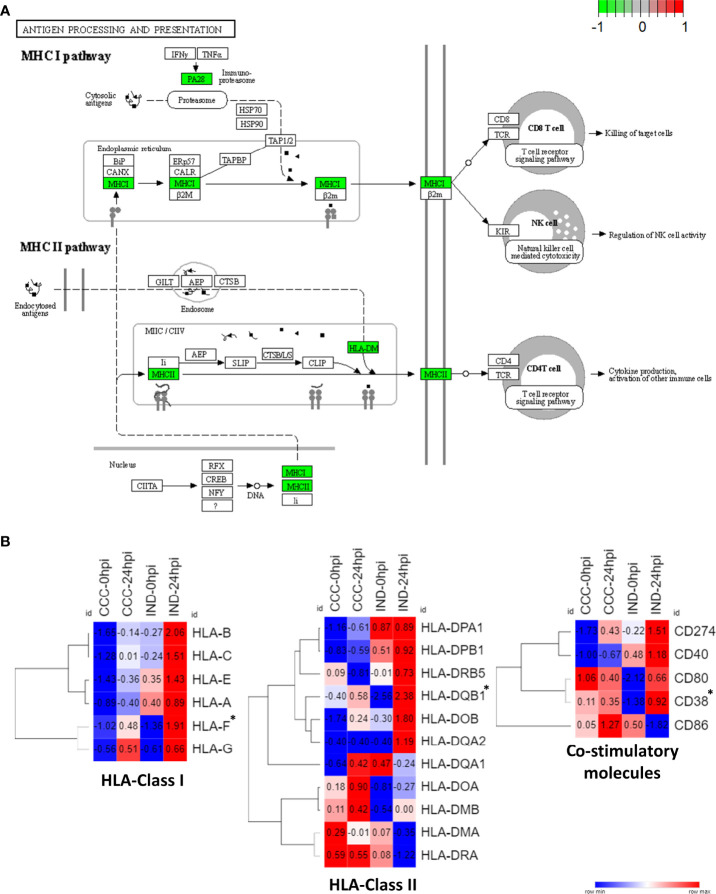
HLA expression pathways in IND and CCC cardiomyocytes. **(A)** DEGs were plotted against KEGG antigen processing and presentation pathway and both MHC I and MHC II pathways had a positive association with IND (green – IND association; red – CCC association). **(B)** Heat maps showing the normalized expression of HLA Class I and Class II genes across all conditions. *DEGs with statistical significance.

Among significant DEGs associated to the IND group there were two MHC-coding genes: *HLA-F* (class I) and *HLA-DBQ1* (class II). Furthermore, CD38, a molecule involved in attachment and T cell engagement was also associated. No HLA coding genes were significantly associated with the CCC group. Interestingly, the expression of HLA class II molecules, which is preferentially expressed by professional antigen-presenting cells (pAPCs), was previously reported in PSC-derived cardiomyocytes after IFN-γ induction (Didié et al., 2017). Thus, we decided to check the expression of major HLA I and II coding genes and the main co-stimulatory molecules across conditions (**Figure 6B**). Of all HLA-related genes with positive expression in our dataset, all classical HLA-class I molecules were majorly upregulated in IND cardiomyocytes at 24 hpi while both groups presented upregulation of HLA-class II genes, although no gene was upregulated in both groups. Along with CD38, CD274 (PD-L1) and CD40 also presented a higher expression in the IND group after infection although no significance was observed. The KEGG plot of antigen processing and presentation (**Figure 6A**) shows that both class I and II MHC branches are exclusively associated to IND group DEGs (negative fold change). Two chemokine genes were significantly associated with the IND group: *CXCL10* and *CXCL11*. Both bind to the CXCR3 receptor on the cell surface of activated T lymphocytes and NK cells.

## Discussion

The effects of *T. cruzi* infection on host cells have been of major interest and have been studied in several cellular models using HeLa, HUVEC, LLC-MK2, PBMCs, murine and primary human cardiomyocytes, among many others (Duran-Rehbein et al., 2014). One of the main obstacles to understand the transcriptional remodeling of human target tissues, especially in the case of patients with severe organ impairment, is the lack of non-invasive techniques to obtain viable tissues for experimentation. Recently, hiPSC-CM were shown to be a feasible platform to investigate *T. cruzi* infection allowing quick responses regarding the infective behavior of the parasite in human cardiac cells (Bozzi et al., 2019), as well as in the screening of drugs or molecules that can influence its infective and proliferative capacity (da Silva Lara et al., 2018; Sass et al., 2019). Here, we successfully generated hiPSC-CM from chronic ChD patients to study the dynamics of *T. cruzi* invasion and proliferation. Our model was able to replicate the molecular effects of early infection in both asymptomatic and individuals with end-stage cardiomyopathy and, despite the small RNA-seq sample size, we could detect important changes in the transcriptional host response in a patient-specific scenario. Although it is known that the clinical outcomes are also influenced by the parasite strain and that using patient-specific strains would be ideal for establishing a reinfection model, we opted to exclusively use the Y strain of *T. cruzi* due to two main reasons: First, the Y strain was isolated in Brazil and it is a member of the TcII discrete type unit (DTU) which was reported as the most frequent in Brazilian patients (Nielebock et al., 2020). Second, because of its application in other important studies tracing infective/proliferative behavior and the host transcriptional changes during the infection of hiPSC-CM (da Silva Lara et al., 2018; Bozzi et al., 2019).

Using the modified Y strain and specific labeling of PBMCs we could efficiently distinguish the rate of infection and parasite replication in monocytes, T and B lymphocytes from both IND and CCC patients. Although no group differences were found, by comparing the *in vitro* infection rates of T and B lymphocytes in our model with previous works (Velge et al., 1991; Souza et al., 2004), we confirmed the low rate of infection in these cell types, but now showing that this rate is maintained up to 48 hpi without a significant increase (**Figure 1A**), and also confirming the ability of *T. cruzi* to proliferate within these cells (**Figure 1B**).

The rate of monocyte infection reported in the literature is variable, since the protocols vary in the hpi curve and in the cells preparation, hindering the direct comparison of our results (Souza et al., 2004; Souza et al., 2007). Some groups choose to infect only adherent monocytes without the presence of other PBMCs, not exploring the effects of the presence of other cells at the time of infection. Still, data on monocyte infection indicate that *T. cruzi* has a high capacity to infect these cells, and at just 3 hpi the infection rate varies between 29% and 80% (Duran-Rehbein et al., 2014). Our results show that at 3 hpi the preferential infection is also for monocytes, with a significant increase in the infection rate in the subsequent hours, indicating that the presence of other PBMCs in the medium does not seem to alter the initial preference of *T. cruzi*. The professional phagocytic role of monocytes may also be a contributing factor to higher rates of infection at 3 hpi since monocytes are activated through *T. cruzi* molecular patterns, as well as to the sharper drop in the number of cells analyzed over time. In addition, although there is a decrease in the number of monocytes over time, the 48/24 hpi GFP intensity ratio (**Figure 1C**) shows that the replication efficiency of the parasite in monocytes is greater than in T and B lymphocytes, indicating that this type of cell is more conductive to parasitic replication. This last observation is intriguing, taken the known role of these cells in parasite clearance. Further studies aiming at a better understanding of the role of monocytes in early *T. cruzi* infection are warranted.

Interestingly, similar to what was seen in PBMCs, no significant differences were observed between IND and CCC hiPSC-CM when infected with *T. cruzi* (**Figure 2B**). The fact that the efficiency of infection and replication in cardiomyocytes is equal in both groups anticipates that the phenotypic outcomes are unlikely to be a matter of parasitic burden. In view of these results, globally exploring the transcriptional response to infection becomes a feasible option to identify transcriptional responses that could shed light on the molecular strategy adopted to overcome the intracellular disturbances caused by infection and to avoid the parasite segregation in the heart tissue.

Differences in the profile of innate immune response and cardiac function were already described when comparing IND and CCC patients. Results from plasma quantification revealed that IND patients present higher levels of serum IL-10 and IL-17A while CCC patients present higher levels of pro-inflammatory IL-6, TNF-α and IFN-γ correlated with impaired LV ejection (Sousa et al., 2017). Interestingly, the same pattern of cytokine expression was also seen in blood monocytes from IND and CCC patients when infected *in vitro* with *T. cruzi* (Souza et al., 2004; Sousa et al., 2014). Studies suggest that INF-γ and TNF-α would be the key mediators of a pro-inflammatory scenario that would lead CCC individuals to develop cardiac tissue injury and that a distinct cytokine landscape upon *T. cruzi* infection would be the reason for IND patients to remain asymptomatic (Chevillard et al., 2018). Transcriptomic analysis of cardiac biopsies from end-stage CCC patients revealed INF-γ as the main expressed cytokine, responsible for triggering the expression of several inflammatory genes (Cunha-Neto et al., 2005). This unbalanced scenario could at least in part explain why these patients present more cardiac tissue injury, with the presence of leukocyte infiltration and fibrosis.

Interestingly, the interferon response appeared in our results as a highly sensitive driver in the cellular response to infection with a considerable fraction of genes differently modulated between groups associated to this pathway. Both type I and II IFN cascades are mediated by TLR recognition in the cell and phagosome membranes and were shown to be crucial in the control of *T. cruzi* replication (Koga et al., 2006). In the GSEA the IFN signaling was statistically associated with the IND group which presented a highly homogeneous IFN-related gene expression at 24 hpi (**Figure 5**).

The triggering of IFN-inducible genes in infected cardiomyocytes is crucial to parasite proliferation control, however the shifting from a protective to a deleterious effect is elusive (Ferreira, 2014). Our results point to a protective role of IFN-inducible genes in IND hiPSC-CM in the first 24 hpi, in which a timely and well-orchestrated response to *T. cruzi* infection seemed crucial to establish an efficient crosstalk between innate and adaptive immune response. Accordingly, *HLA-F* (class I) and *HLA-DQB1* (class II) were significantly associated with IND in GSEA results, and all HLA Class I genes were upregulated in this group at 24 hpi (**Figure 6B**). Of note, *HLA-DQB1* gene was previously shown to confer protection against cardiac or digestive disease in Brazilian chagasic patients (Deghaide et al., 1998). Other molecules such as CD38 (significantly upregulated), CD247, CD40 and CD80 were also upregulated in the IND groups at 24 hpi, establishing a proper scenario for leukocyte stimulation and activation. Importantly, CD247 (PD-L1) is known to be a crucial immunological checkpoint involved in the immune regulation of T cells and the treatment with checkpoint inhibitors led to increased leukocyte infiltration and heart damage in *T. cruzi* infected mice (Fonseca et al., 2018).

Interestingly, the expression of ECM-related genes was sensitive to infection in IND cardiomyocytes while remained unchanged in CCC. At early moments of infection, *T. cruzi* is known to interact with components of ECM inducing matrix remodeling and some of the genes associated to “EM transition” (Hallmark) and “ECM organization” (Reactome) are part of the proposed *T. cruzi*-ECM interactome (*MMP2*/*COL1A2*/*COL6A2*/*COL5A1*) regulated by the parasite molecule gp38 (Nde et al., 2012). The regulation of both pro-fibrotic and anti-fibrotic genes was shown in primary human cardiomyocytes at early moments of infection, indicating that cardiac cells are able to respond to the parasite-induced production of ECM components (Udoko et al., 2016). Thus, the downregulation of ECM-related genes by IND cardiomyocytes might indicate a better control in the expression of ECM-remodeling genes, protecting cells from acquiring a pro-fibrotic phenotype.

We could also detect pathways that work in opposite directions after infection. Both mTORC1 signaling and UPR were upregulated in CCC but downregulated in IND at 24 hpi (**Figure 5**). The mTORC1 signaling is crucial in early moments of infection, as shown by Libisch et al. (2018) (Libisch et al., 2018). Briefly, the authors observed that activation of mTORC1 signaling upon infection in human cardiomyocytes induces mitochondrial biogenesis and increased reactive-oxygen species (ROS) production leading to oxidative damage and the development a pathological phenotype. Also, ROS-mediated ER stress is a known phenomenon which causes the misfolding of proteins and activation of UPR (Cao and Kaufman, 2014). Some of the molecules triggered by UPR induce apoptotic signaling, which is the case of CCAAT/enhancer-binding protein (C/EBP) transcription factors. Here, the *CEBPG* gene was upregulated in CCC as result of infection. The knockdown of *CHOP*, a main C/EBP family member highly active under ER stress, in a murine model of cardiac overload resulted in less cardiac dysfunction indicating that C/EBP transcription factors are key regulators of cardiomyocyte apoptosis under pathological stress (Wang et al., 2021).

The production of ROS in infected cells is understood as an active response to parasite elimination, but with the consequence of generating damage to the myocardium as shown in animal models of CCC (Paiva et al., 2018) and also favoring the parasite’s survival and causing DNA damage to the host (Florentino et al., 2021). In CCC hiPSC-CM, the activation of the mTORC1 pathways indicates that ROS response is the main strategy for these cells to avoid *T. cruzi* proliferation but with consequences to the correct function to the translational machinery of the cell, leading to the activation of ER-stress response and consequent apoptosis, generating an unfavorable scenario for the functioning and survival of the cell.

## Conclusion

Although our model does not fully recapitulates the chronic aspects of CCC nor the interplay among the several discrete typing units (DTUs) of *T. cruzi* and innate immune cells and hiPSC-CM, our results point to a differential landscape in which infected IND cardiomyocytes exhibit a favorable immunological profile that promptly upregulates molecules involved in the innate-adaptive crosstalk, leading to a better orchestration of antigen processing and presentation as well as the downregulation of stress-related genes that could lead to oxidative stress and cardiomyocyte damage. On the other hand, an unbalanced homeostatic profile at 24 hpi disfavors the control of infection-induced ROS and ER-stress in CCC hiPSC-CM that superimposes its ability to develop a specific response to the parasite which in turn results in a long-term dependence of a primary IFN-mediated response. In conclusion, baseline genetic differences modulate group-specific responses to infection which may impact in the presentation of different clinical outcomes.

## Data Availability Statement

The datasets presented in this study can be found in online repositories. The name of the repository and accession number can be found below: GEO, NCBI; GSE203525.

## Ethics Statement

The studies involving human participants were reviewed and approved by Comissão de Ética para Análise de Projetos de Pesquisa – CAPPesq - Hospital das Clínicas da Faculdade de Medicina da USP – CAAE: 89242218.0.0000.0068. The patients/participants provided their written informed consent to participate in this study.

## Author Contribution

TO and AP conceptualized and designed the study, analyzed data, and wrote the manuscript. CD and ES assisted the conceptualization and design of the study. GV designed experiments and assisted data analysis. JA and LF performed experiments. JS, CS, JK and AP assisted with funding and final manuscript elaboration. All authors contributed to the article and approved the submitted version.

## Funding

This work was supported by Conselho Nacional de Pesquisa - CNPq (#420168/2016-8) and Fundação de Amparo à Pesquisa do Estado de São Paulo – FAPESP (#17/20593-7, #17/13706-0 and #19/11821-1). AP, CS and JS received funding for this study from NHLBI R01HL133165.

## Conflict of Interest

The authors declare that the research was conducted in the absence of any commercial or financial relationships that could be construed as a potential conflict of interest.

## Publisher’s Note

All claims expressed in this article are solely those of the authors and do not necessarily represent those of their affiliated organizations, or those of the publisher, the editors and the reviewers. Any product that may be evaluated in this article, or claim that may be made by its manufacturer, is not guaranteed or endorsed by the publisher.
