# Supplementary material for: Different Transcriptomic Response to T. cruzi Infection in hiPSC-Derived Cardiomyocytes From Chagas Disease Patients With and Without Chronic Cardiomyopathy
Source: Front Cell Infect Microbiol. 2022 Jul 7;12:904747. doi: 10.3389/fcimb.2022.904747 (PMC9301326; doi:10.3389/fcimb.2022.904747)
Supplement: Supplementary file 1 [file DataSheet_1.docx]

Supplementary Material

# Supplementary Figures


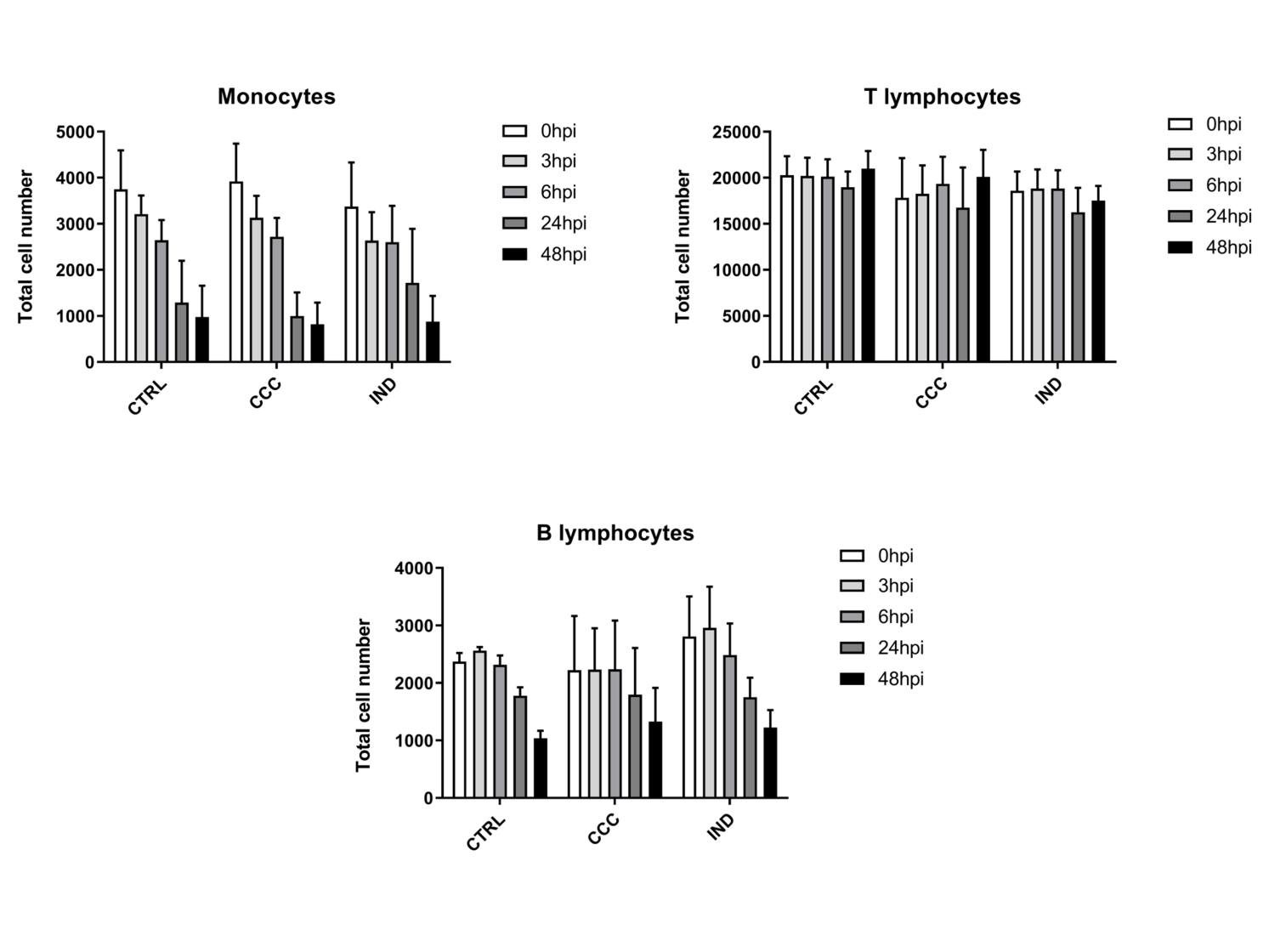


**Supplementary Figure S1 -** Total number of Monocytes, T Lymphocytes and B Lymphocytes analyzed across the curve of infection.


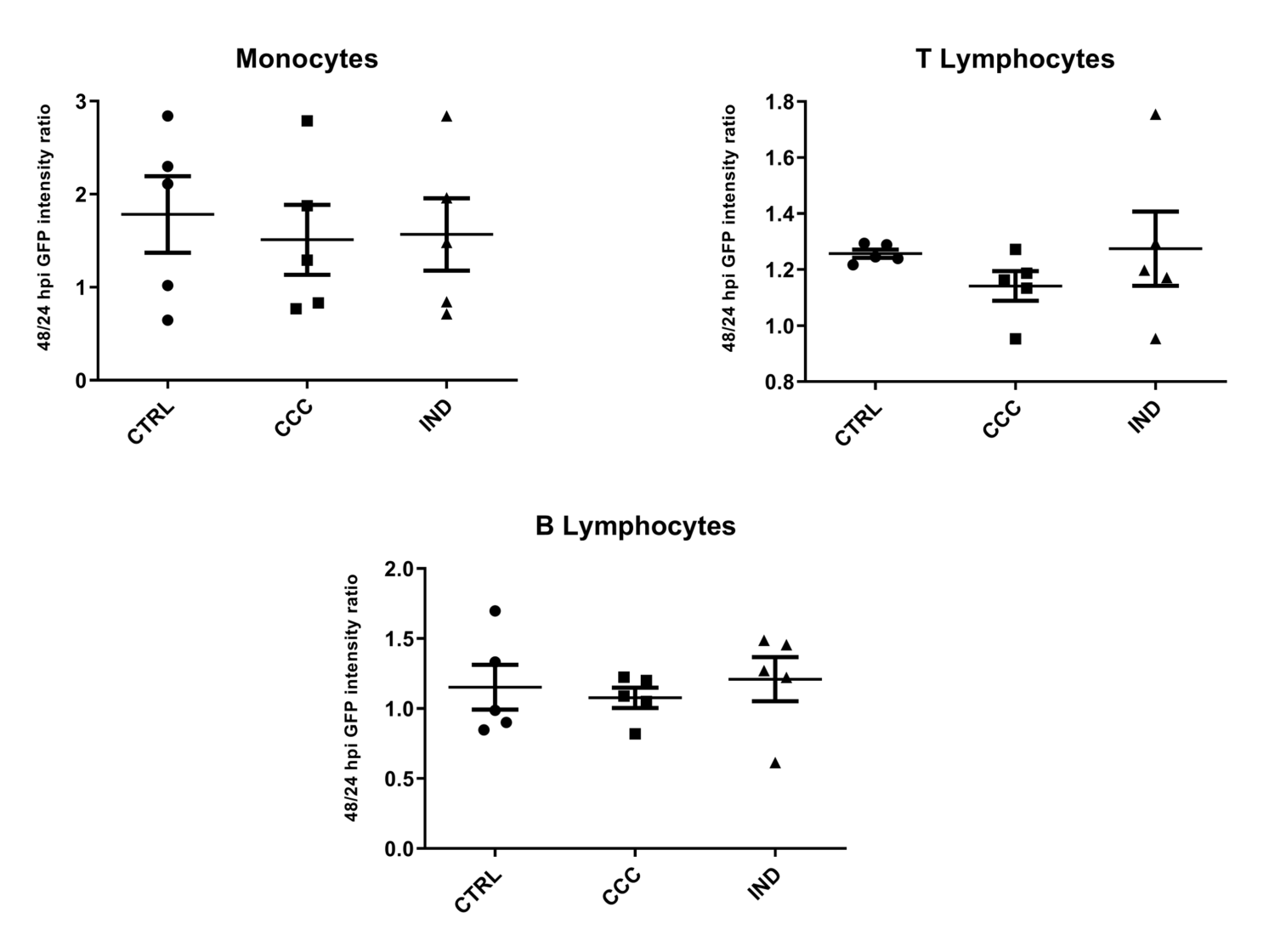
**Supplementary Figure S2 -** 48/24 hpi GFP intensity ratio separated by cell type.


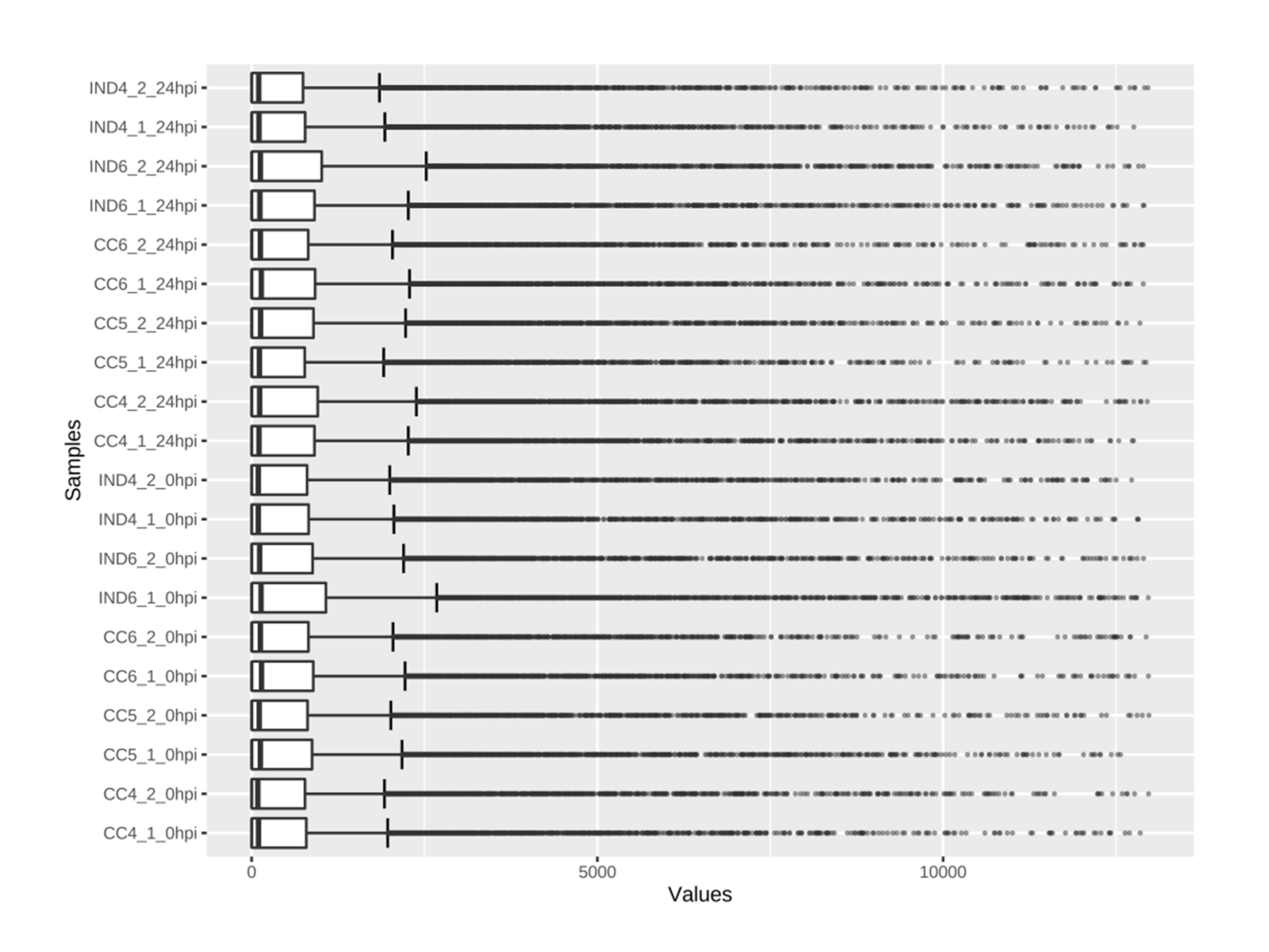


**Supplementary Figure S3 -** Count distribution of replicates.


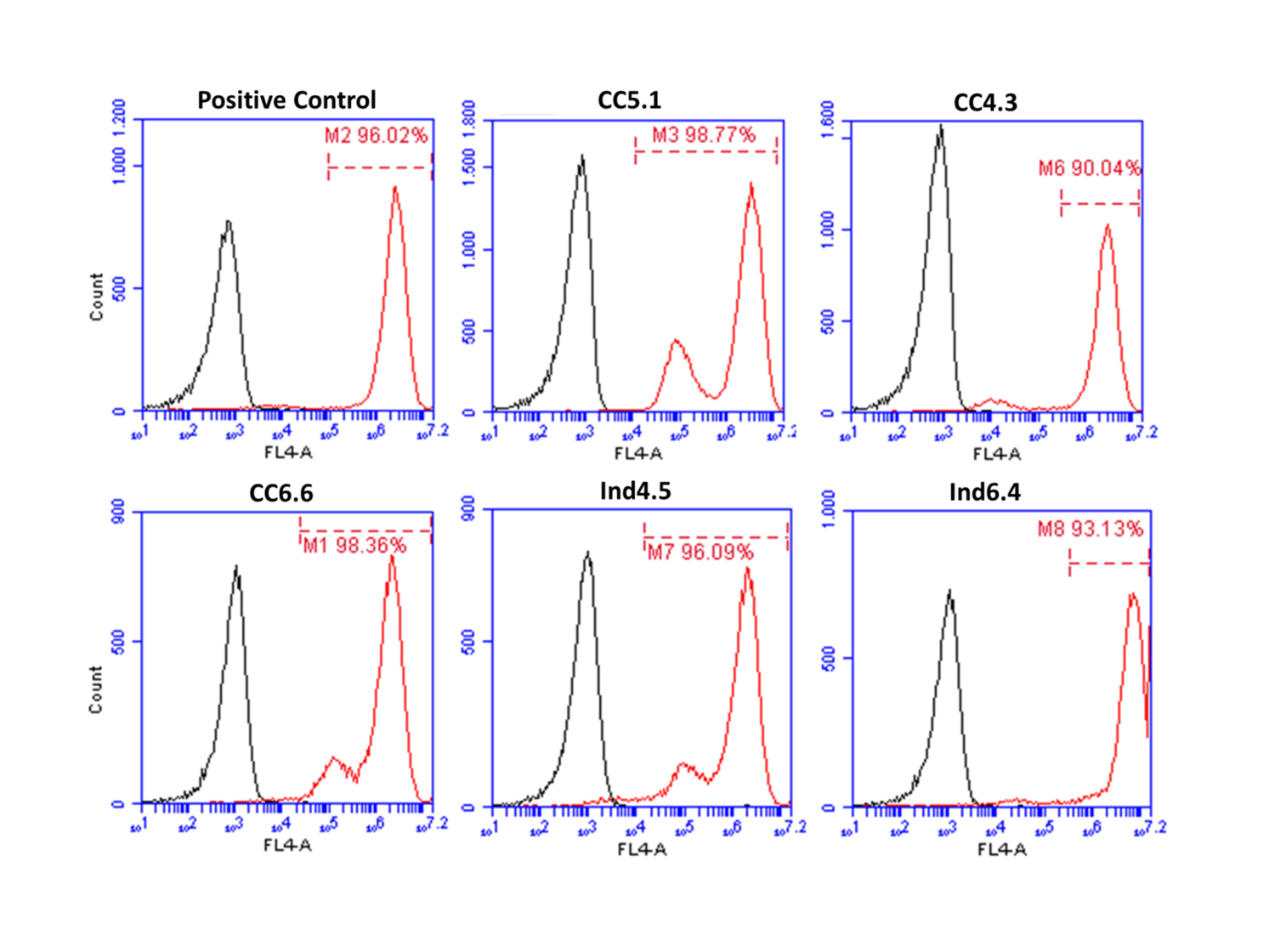


**Supplementary Figure S4 -** Quantification of Cardiac Troponin I in hiPSC-CM by flow cytometry.


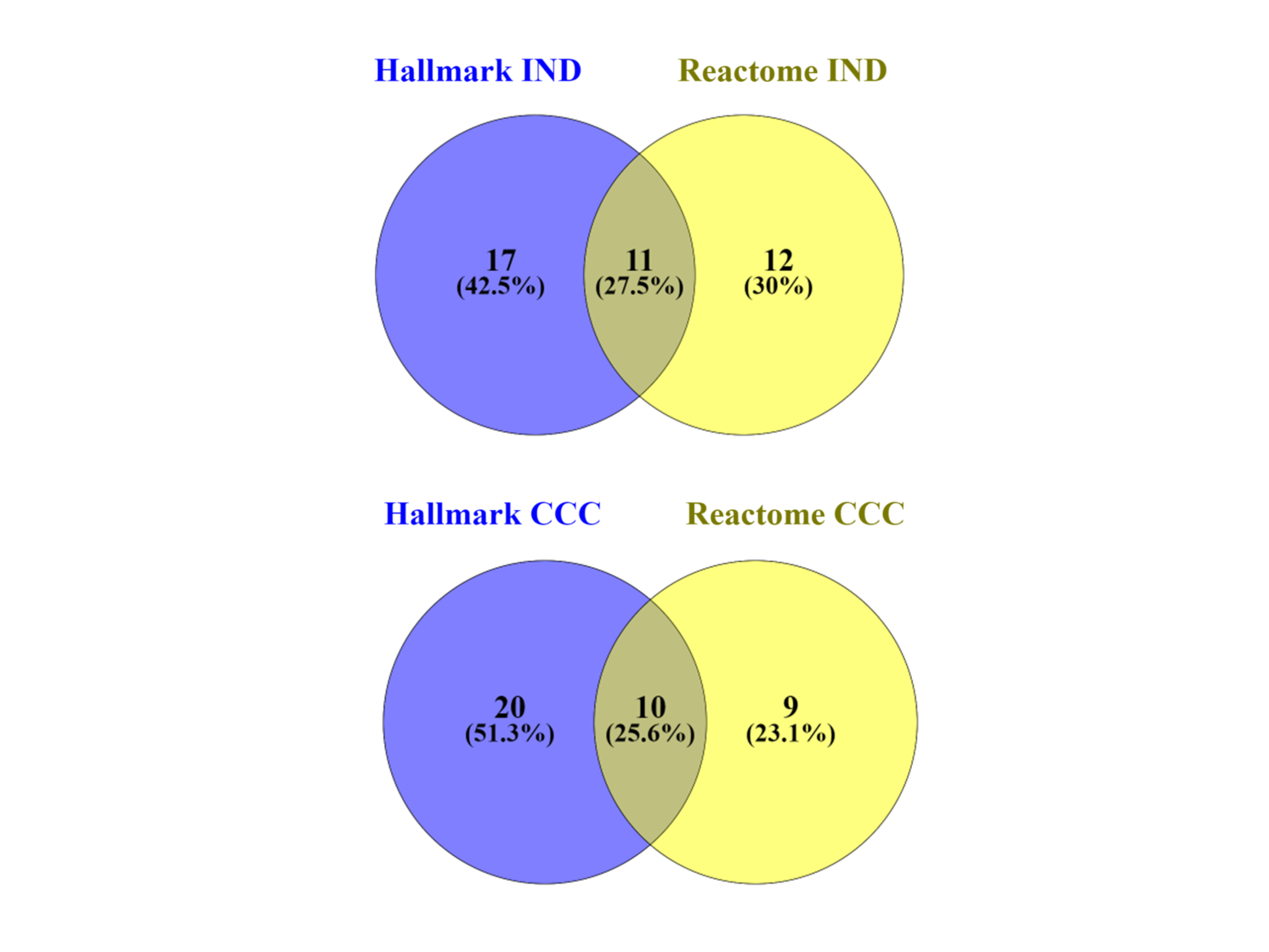
**Supplementary Figure S5 -** Number of genes associated to pathways in Hallmark and Reactome GSEA with IND and CCC DEGs


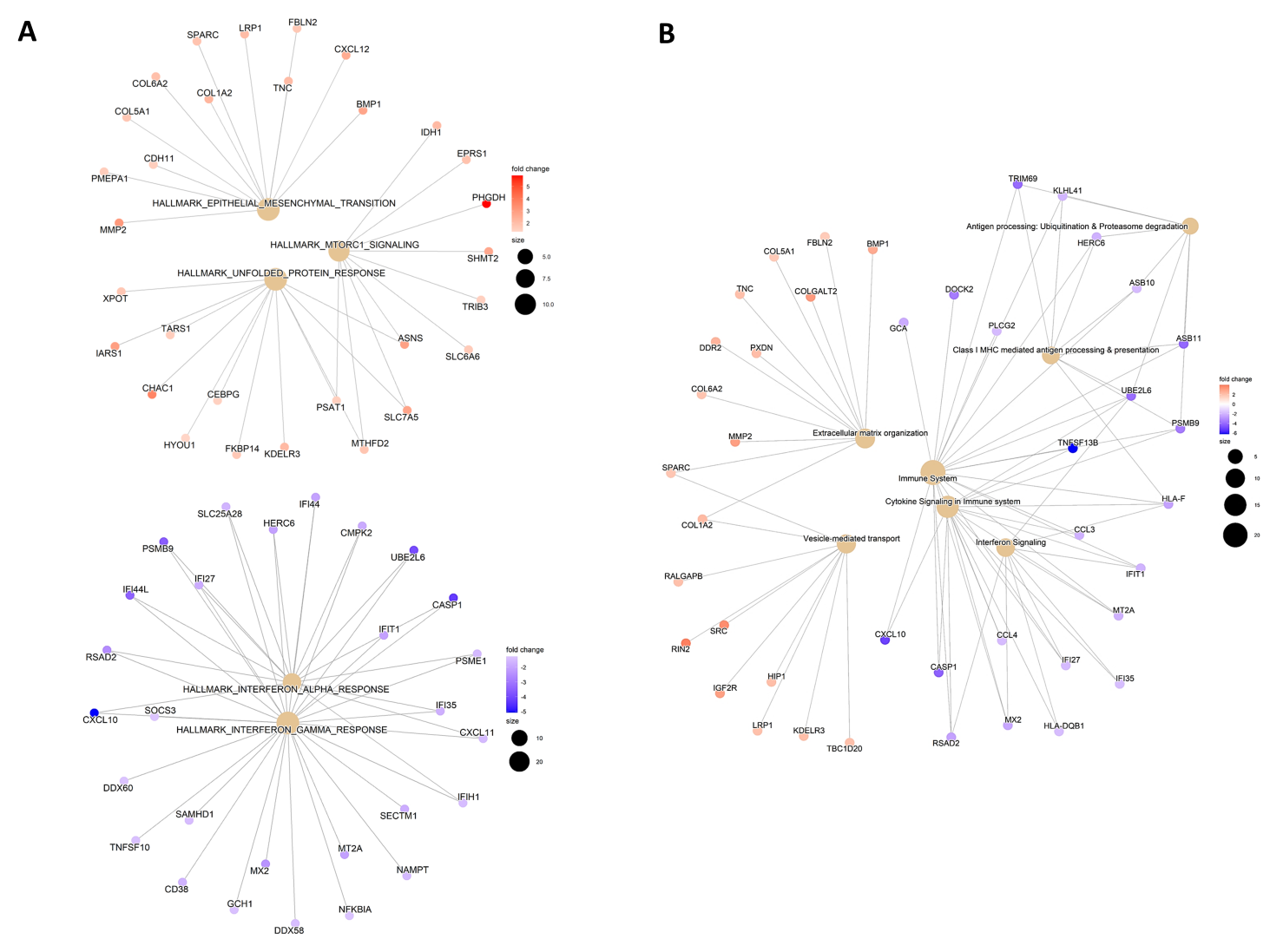


**Supplementary Figure S6** - Network plots showing the connection of leading edge genes in (A) Hallmark (Up – CCC, Bottom – IND) and (B) Reactome analysis (Red dots – CCC; Blue Dots – IND)

.

# 2. Supplementary Table

**Supplementary Table S1 -** Sex, age and main clinical features (CCC) of patients included in the study

| **Patient ID** | **Sex** | **Age** | **Main Clinical Features** |
| --- | --- | --- | --- |
| **CCC** | | | |
| **CCC1** | F | 57 | Inferolateral wall hypokinesia; VEF 55% |
| **CCC2** | M | 33 | VEF 19%; VD and VE dilatation and hypokinesis; diffuse fibrosis; CPA history |
| **CCC3** | M | 49 | VEF 48% and frequent polymorphic VE |
| **CCC4** | M | 45 | Stroke history; Repolarization alterations; Descreased septal contracting forces; paroxysmal AF; VEF 49% |
| **CCC5** | F | 51 | VEF 46%; apical region dyskinesia |
| **CCC6** | F | 48 | VEF 42%; Diffuse hipokinesia; LV eccentric hypertrophy; syncope history |
| **IND** | | | |
| **IND1** | F | 75 | No chronic chagas-related symptoms |
| **IND2** | M | 48 | No chronic chagas-related symptoms |
| **IND3** | M | 64 | No chronic chagas-related symptoms |
| **IND4** | M | 62 | No chronic chagas-related symptoms |
| **IND5** | M | 68 | No chronic chagas-related symptoms |

Supplementary Table S2 - Full list of gene sets enriched in Hallmark GSEA analysis regardless of padj significance.

| **GeneSet** | **ES** | **NES** | **p-value** | **FDR** | **size** | **LEG** | **Core Enrichment** |
| --- | --- | --- | --- | --- | --- | --- | --- |
| **IND-associated gene sets** | | | | | | | |
| **INTERFERON_GAMMA_RESPONSE** | **-0.627** | **-3.31** | **0.002** | **0.0221** | **36** | **28** | **SOCS3/NFKBIA/DDX60/GCH1/TNFSF10/DDX58/SAMHD1/IFIH1/CXCL11/**  **PSME1/NAMPT/SLC25A28/CD38/IFI35/SECTM1/IFI27/CMPK2/IFI44/IFIT1**  **/HERC6/MT2A/MX2/RSAD2/IFI44L/PSMB9/UBE2L6/CASP1/CXCL10** |
| **INTERFERON_ALPHA_RESPONSE** | **-0.684** | **-3.01** | **0.002** | **0.0221** | **21** | **15** | **IFIH1/CXCL11/PSME1/SLC25A28/IFI35/IFI27/CMPK2/IFI44/HERC6/RSAD2**  **/IFI44L/PSMB9/UBE2L6/CASP1/CXCL10** |
| **KRAS_SIGNALING_DN** | **-0.589** | **-1.90** | **0.004** | **0.0612** | **9** | **7** | **ARPP21/CKM/IFI44L/NR6A1/RSAD2/SHOX2/SNCB** |
| **KRAS_SIGNALING_UP** | **-0.401** | **-1.45** | **0.087** | **0.4516** | **13** | **3** | **CA2/CXCL10/DOCK2** |
| **IL6_JAK_STAT3_SIGNALING** | **-0.498** | **-1.43** | **0.116** | **0.3979** | **7** | **3** | **CD38/CXCL10/CXCL11** |
| **COMPLEMENT** | **-0.337** | **-1.40** | **0.100** | **0.3695** | **19** | **11** | **ADRA2B/APOBEC3F/APOBEC3G/CA2/CASP1/CD55/GCA/L3MBTL4/LAP3**  **/LGALS3/PSMB9** |
| **MITOTIC_SPINDLE** | **-0.415** | **-1.36** | **0.132** | **0.3738** | **10** | **4** | **ARHGAP27/ARHGEF3/DOCK2/LRPPRC** |
| **UV_RESPONSE_UP** | **-0.350** | **-1.32** | **0.157** | **0.3949** | **14** | **12** | **CA2/E2F5/ENO2/GCH1/HLA-F/IRF1/NFKBIA/PPIF/RET/SHOX2/SOD2/TAP1** |
| **OXIDATIVE_PHOSPHORYLATION** | **-0.516** | **-1.27** | **0.186** | **0.4322** | **5** | **2** | **ATP1B1/LRPPRC** |
| **IL2_STAT5_SIGNALING** | **-0.320** | **-1.21** | **0.221** | **0.4294** | **14** | **4** | **CA2/CDCP1/CXCL10/LRRC8C** |
| **CCC-associated gene sets** | | | | | | | |
| **EPITHELIAL_MESENCHYMAL_TRANSITION** | **0.548** | **2.130** | **0.002** | **0.0221** | **19** | **12** | **MMP2/BMP1/CXCL12/COL1A2/TNC/LRP1/COL6A2/SPARC/COL5A1**  **/FBLN2/PMEPA1/CDH11** |
| **UNFOLDED_PROTEIN_RESPONSE** | **0.547** | **2.21** | **0.002** | **0.0221** | **13** | **12** | **CHAC1/IARS1/ASNS/SLC7A5/KDELR3/MTHFD2/XPOT/FKBP14/TARS1**  **/PSAT1/CEBPG/HYOU1** |
| **MTORC1_SIGNALING** | **0.530** | **1.96** | **0.005** | **0.0391** | **15** | **10** | **PHGDH/ASNS/SLC7A5/SHMT2/IDH1/EPRS1/MTHFD2/SLC6A6/PSAT1/TRIB3** |
| **MYOGENESIS** | **0.413** | **1.57** | **0.053** | **0.2036** | **18** | **6** | **APLNR/COL6A2/EPHB3/IGF1/PLXNB2/SPARC** |
| **APICAL_JUNCTION** | **0.410** | **1.44** | **0.085** | **0.3144** | **14** | **8** | **BMP1/CDH11/DLG1/MAPK14/MMP2/MYH10/PCDH1/SRC** |
| **GLYCOLYSIS** | **0.424** | **1.36** | **0.132** | **0.378** | **11** | **6** | **CHPF/CHPF2/COL5A1/HDLBP/IDH1/KDELR3** |
| **XENOBIOTIC_METABOLISM** | **0.364** | **1.25** | **0.207** | **0.4808** | **13** | **4** | **IDH1/IGF1/PYCR1/SHMT2** |
| **UV_RESPONSE_DN** | **0.363** | **1.22** | **0.214** | **0.4671** | **13** | **6** | **COL1A2/DLG1/FYN/KALRN/MAPK14/SLC7A1** |
| **NOTCH_SIGNALING** | **0.474** | **1.15** | **0.286** | **0.5193** | **5** | **3** | **HEYL/JAG1/MAML2** |
| **ADIPOGENESIS** | **0.393** | **1.01** | **0.418** | **0.5429** | **6** | **6** | **ALDH2/COL15A1/DNAJC15/IDH1/PTGER3/SCARB1** |
